# Melioidosis fatalities in captive slender-tailed meerkats (*Suricata suricatta)*: combining epidemiology, pathology and whole-genome sequencing supports variable mechanisms of transmission with one health implications

**DOI:** 10.1186/s12917-019-2198-9

**Published:** 2019-12-19

**Authors:** Audrey Rachlin, Cathy Shilton, Jessica R. Webb, Mark Mayo, Mirjam Kaestli, Mariana Kleinecke, Vanessa Rigas, Suresh Benedict, Ian Gurry, Bart J. Currie

**Affiliations:** 1 Menzies School of Health Research, Charles Darwin University, Darwin, Casuarina NT 0811 Australia; 2 Department of Primary Industry and Resources, Berrimah Veterinary Laboratory, Berrimah Farm, Makagon Road, Berrimah, Northern Territory 0828 Australia; 3 Research Institute for the Environment and Livelihoods, Charles Darwin University, Darwin, Northern Territory 0811 Australia; 4 Parap Veterinary Hospital, Parap, Darwin, Northern Territory 0820 Australia; 5 Royal Darwin Hospital and Northern Territory Medical Program, Darwin, Northern Territory 0811 Australia

**Keywords:** *Burkholderia pseudomallei*, Melioidosis, Northern Australia, Whole-genome sequencing, Source tracing, Slender-tailed meerkats, Outbreak

## Abstract

**Background:**

Melioidosis is a tropical infectious disease which is being increasingly recognised throughout the globe. Infection occurs in humans and animals, typically through direct exposure to soil or water containing the environmental bacterium *Burkholderia pseudomallei*. Case clusters of melioidosis have been described in humans following severe weather events and in exotic animals imported into melioidosis endemic zones. Direct transmission of *B. pseudomallei* between animals and/or humans has been documented but is considered extremely rare. Between March 2015 and October 2016 eight fatal cases of melioidosis were reported in slender-tailed meerkats (*Suricata suricatta)* on display at a Wildlife Park in Northern Australia. To further investigate the melioidosis case cluster we sampled the meerkat enclosure and adjacent park areas and performed whole-genome sequencing (WGS) on all culture-positive *B. pseudomallei* environmental and clinical isolates.

**Results:**

WGS confirmed that the fatalities were caused by two different *B. pseudomallei* sequence types (STs) but that seven of the meerkat isolates were highly similar on the whole-genome level. Used concurrently with detailed pathology data, our results demonstrate that the seven cases originated from a single original source, but routes of infection varied amongst meerkats belonging to the clonal outbreak cluster. Moreover, in some instances direct transmission may have transpired through wounds inflicted while fighting.

**Conclusions:**

Collectively, this study supports the use of high-resolution WGS to enhance epidemiological investigations into transmission modalities and pathogenesis of melioidosis, especially in the instance of a possible clonal outbreak scenario in exotic zoological collections. Such findings from an animal outbreak have important One Health implications.

## Background

Melioidosis is a disease of significant public health importance throughout much of the tropics, most notably in Southeast Asia and Northern Australia where it is considered highly endemic in both humans and animals [1]. The majority of cases arise through direct percutaneous exposure to the infections aetiological agent, *Burkholderia pseudomallei* [2], though case reports associated with severe weather events [3] and contaminated drinking supplies [4] have implicated inhalation and ingestion as potentially significant sources of infection. Recent studies from Thailand have raised the possibility that ingestion of water contaminated with *B. pseudomallei* may be a more common infecting event than previously thought, especially in endemic regions with unchlorinated water supplies [5]. Disease severity and clinical manifestations vary widely and in addition to route of infection, are influenced by host susceptibility, infecting bacterial dose and differential *B. pseudomallei* strain virulence [6, 7].

In humans melioidosis is being increasingly recognised as an opportunistic infection, with severe disease and death very uncommon in a healthy human host provided there is timely diagnosis and access to appropriate antibiotics [8]. In animals, the disease has now been documented in over 50 species [9, 10] with notable variations in disease susceptibility [9, 11, 12]. Animals native to melioidosis-endemic regions are generally resistant to disease despite frequent exposure to *B. pseudomallei* in the environment, while many exotic animals imported into zoos in endemic regions are especially susceptible and develop severe and often rapidly fatal disease [9, 11–14]. Melioidosis has been reported in a wide-array of livestock and in domestic pets [9, 10, 12]. Surveillance from tropical Northern Australia and Thailand has also shown that sheep [15], camels [16] and alpacas [17] readily succumb to melioidosis, while goats have diverse presentations similar to humans [12, 18] and pigs can often have asymptomatic internal infections [19].

Although zoonotic transmission of *B. pseudomallei* and direct transmission between animals is considered rare, outbreaks related to exposure of a contaminated environmental source have been reported in both endemic and non-endemic settings, including piggeries in Queensland, Australia [19], non-human primates in European and Asian zoos [13, 20], and previous incidents in captive slender-tailed meerkats on display in eastern Thailand [21]. Regular outbreaks observed in intensive livestock farms [19] and in zoos [14] suggest that stressful conditions may initiate the onset of disease, particularly in exotic non-native species.

High-resolution molecular fingerprinting tools have recently been used to investigate melioidosis case cluster and outbreak scenarios in both humans and animals [22–25]. Multi-locus sequence typing (MLST) is a popular, widely applicable genotyping tool with a global online database for assessing *B. pseudomallei* strain relatedness and melioidosis source attribution (http://pubmlst.org/bpseudomallei) [26–28]. Despite this, there are instances where MLST is unable to determine fine-scale population structures due to exceedingly high rates of recombination in the *B. pseudomallei* genome [29, 30]. Phylogenetic reconstruction of *B. pseudomallei* populations using whole-genome sequencing (WGS) data can provide a highly robust method of outbreak source attribution and strain tracing on both an inter and intra-continental level [31–33]. Such technology is also highly applicable should a biothreat scenario arise involving this tier 1 select agent (http://www.selectagents.gov/). Additionally, WGS is now being used to investigate the genomic basis of differential virulence amongst *B. pseudomallei* isolates [7].

In this study, WGS and comparative genomic analysis were used to examine a cluster of eight fatal melioidosis cases reported in imported African slender-tailed meerkats (*Suricata suricatta)* on display at a Wildlife Park in Darwin, Northern Territory (NT), Australia. We undertook six rounds of environmental sampling in relation to the cases and performed MLST and WGS on all culture-positive *B. pseudomallei* environmental and clinical isolates. Used in conjunction with pathology data, we established the most likely modes of infection and transmission responsible for the outbreak.

## Results

### Pathology and bacterial culture

**Fig. 1:**
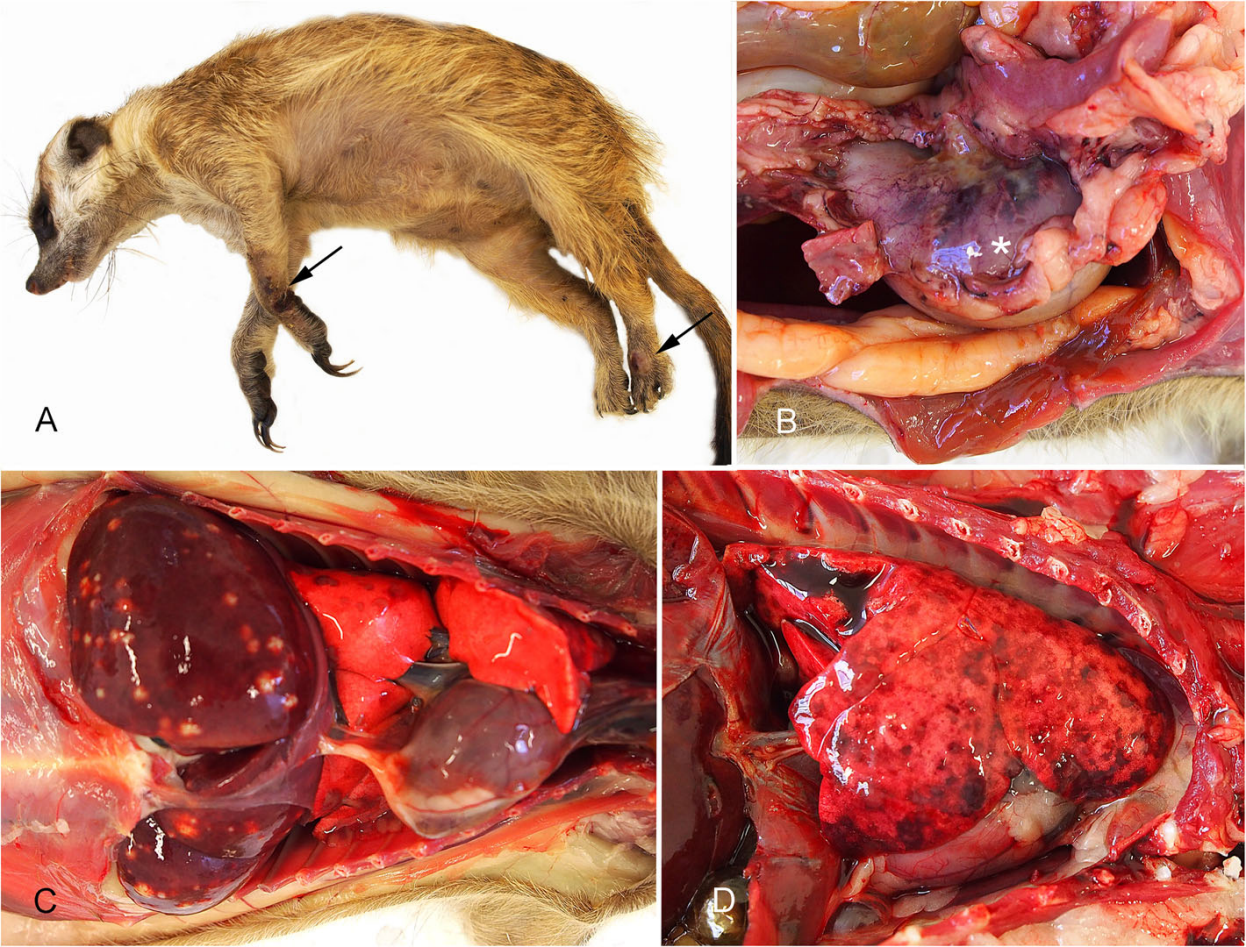
Gross pathology of melioidosis in meerkats. Except for image A, all meerkats are positioned with the head to the right of the image. (**a**.) Typical ulcerative skin lesions involving left carpus and dorsal aspect of left hind foot (arrows) in case #3. (**b.)** Stomach of case #2 (asterisk) showing severe haemorrhage involving lesser curvature and purulent exudate in adjacent mesentery. (**c**.) Multifocal pale suppurative lesions throughout the liver in case #4. Note also multiple small circular red foci of embolic infection in the lungs. (**d.)** Lung of case #6 with abundant coalescing red foci, many with central pallor, representing severe, diffuse embolic showering of the lung

Significant pathological findings are detailed for each case in Table 1. Five of the meerkats had superficial erosive to deep ulcerative necrotising suppurative skin lesions with associated cellulitis, usually involving multiple sites, commonly including the distal legs in the region of the carpi or tarsi, the scrotum and the tail **(**Fig. 1a**)**. In case #3, #4 and #5, the skin lesions were associated with fibroplasia, and were therefore judged to be the oldest lesions present (at least 3 days duration). In two of these cases, *B. pseudomallei* was cultured from a skin lesion. In case #8, although there was a severe tarsal lesion from which *B. pseudomallei* was cultured, the lesion appeared relatively acute with no associated fibroplasia. In the fifth case with skin lesions (case #6), the lesions were mild and considered likely incidental.

**Table 1:** Meerkat (*Suricata suricatta*) case details

Case No.	Date of death	Sex, Age	Pathology diagnoses	*B. pseudomallei* positive culture^1^	Suspected route of exposure
**1**	Mar. 18, 2015	Male, 3 yrs.	**Liver**: Multifocal intrahepatic portal suppurative thrombosis and random multifocal suppurative hepatitis **Lung**: Single large suppurative focus contiguous with suppurative venous thrombus; random multifocal suppurative pneumonia	Bone marrow (femur), spleen, lung	Ingestion (portal lesion)
**2**	April 23, 2016	Female, 10 mos.	**Stomach**: Transmural haemorrhagic, suppurative gastritis with adjacent cranial mesenteric suppurative peritonitis with fibroplasia **Liver**: Multifocal intrahepatic portal suppurative thrombosis and random multifocal suppurative hepatitis **Lung:** Multifocal suppurative pneumonia, often associated with haemorrhage and septic thrombosis	Cranial mesentery, lung, spleen	Ingestion (gastric and portal lesions)
**3**	April 26, 2016	Male, 10 mos.	**Skin**: Multifocal severe, ulcerative, suppurative deep dermatitis and cellulitis with associated fibroplasia (left forearm and left hind foot). Right scrotum open and draining necrohaemorrhagic exudate **Liver**: Random multifocal suppurative hepatitis **Lung**: Multifocal suppurative pneumonia, variably associated with haemorrhage and septic thrombosis	Left hind foot lesion, lung, spleen	Cutaneous (forearm and hind foot lesions)
**4**	July 13, 2016	Female, 12 mos.	**Skin**: Unhealed distal tail amputation with associated suppurative deep dermatitis, fibrosis and bone remodelling; Focal ulcerative dermatitis (dorsal to left eye); Bilateral dorsal carpal epidermal hyperplasia and hyperkeratosis. **Left stifle**: Suppurative arthritis **Liver and lung**: Random multifocal suppurative hepatitis and pneumonia **Kidney**: Segmental pyelonephritis	Lung, spleen, liver	Cutaneous (tail amputation lesion)
**5**	July 25, 2016	Male, 12 mos.	**Skin**: Multifocal subacute to chronic, ulcerative, suppurative dermatitis (most severely involving the scrotum and perineum, more superficially involving the bridge of nose, above left eye, left ear pinna, tail, dorsal aspects both carpi and all feet, scrotum and perineum) **Liver and lung**: Random multifocal suppurative hepatitis and pneumonia	Lung, spleen positive. Perineal/scrotal swab negative	Cutaneous (multiple chronic skin lesions)
**6**	Sept. 21, 2016	Male, 15 mos.	**Liver**: Multifocal portal suppurative thrombosis with associated fibroplasia **Lung**: Random multifocal suppurative pneumonia **Skin**: Mild multifocal erosive dermatitis (ventral scrotum, medial hind foot)	Liver, lung, spleen	Ingestion (portal lesion)
**7**	Oct. 7, 2016	Female, 3 yrs.	**Intestine:** Segmental transmural necrotising, suppurative enteritis with regional suppurative lymphadenitis **Lumbar vertebral column**: Multifocal suppurative thrombosis and osteomyelitis with extension into vertebral canal **Femur**: Suppurative osteomyelitis extending to periosteum with associated fibrosis **Lung**: Random multifocal suppurative pneumonia	Blood, liver, lung positive. Rectal and throat swabs negative	Ingestion (suppurative enteritis with regional suppurative lymphadenitis)
**8**	Oct. 23, 2016	Female, 16 mos.	**Skin**: Ulcerative, necrotising, suppurative deep dermatitis and cellulitis (left tarsal region) **Vertebral column**: Multifocal suppurative osteomyelitis **Lung**: Random multifocal suppurative pneumonia **Liver**: Extrahepatic portal vein suppurative phlebitis and thrombosis with associated fibroplasia. Liver otherwise too decomposed to assess	Left tarsal lesion swab, liver, lung, rectal swab, throat swab	Ingestion (portal lesion) or cutaneous (tarsal lesion)

Signalment, history, pathological findings and *B. pseudomallei* culture results from the eight meerkat cases. ^1^Unless otherwise specified, the tissues noted as culture positive are all the tissues for which culture was attempted.

**Fig. 2:**
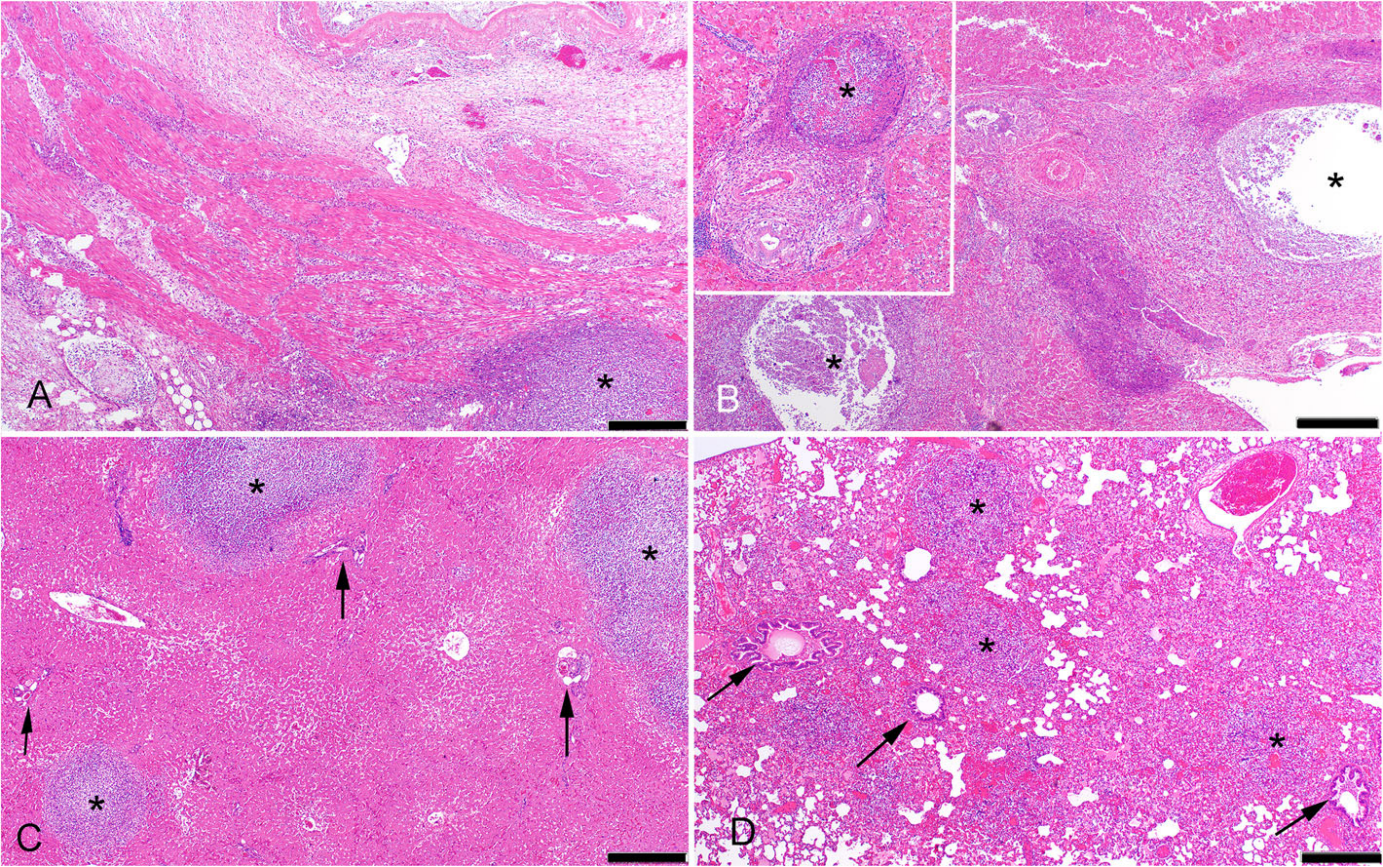
Histopathology of melioidosis in meerkats. All main images are low power (40X) views, bars = 500 μm. (**a.)** Gastric wall of case #2 (also depicted grossly in Fig. 1b). The lumen is at the top right of the image, serosa near the lower left. There is marked congestion and transmural oedema, with vascular thrombosis and foci of necrotic neutrophils in the wall (asterisk). The muscular tunics and serosa are mildly expanded by early fibroplasia. (**b.)** Entrance of portal vein (asterisks indicate two sections of the lumen) at the hepatic hilus in case #6. The architecture of the vein is obliterated by a dense infiltrate of necrotic neutrophils expanding into the surrounding tissue, which is expanded by early fibroplasia. The inset shows a higher magnification of a portal triad with thrombosed intrahepatic tributary of the portal vein largely obliterated by an infiltrate of necrotic neutrophils (asterisk, note portal arteriole and bile duct to lower left of the vein). **(c.)** Liver from case #4 (also depicted grossly in Fig. 1c). Suppurative foci (asterisks) in this case are random, typical of embolic showering of bacteria from the systemic circulation. Note unaffected nearby portal triads (arrows). **(d.)** Embolic showering of the lung in case #6 (also depicted grossly in Fig. 1d) in which the primary lesion was likely via the portal circulation (depicted in Fig. 2b). Multiple random foci of neutrophil infiltration (asterisks) with surrounding haemorrhage. Note relatively unaffected nearby bronchioles (arrows)

The liver exhibited lesions in all meerkats, with two distinct patterns. In four of the cases (cases #1, #2, #6 and #8), extrahepatic and/or intrahepatic portal veins contained thrombi infiltrated with neutrophils, necrosis of the vessel wall and large suppurative foci centred on remains of portal triads **(**Fig. 2b**)**. In cases #6 and #8 there was associated fibroplasia. In case #2, in addition to the suppurative thrombi in portal veins, there was also severe transmural suppurative gastritis and contiguous cranial mesenteric suppurative peritonitis with associated fibroplasia (Figs. 1b, 2a). The second distinct pattern of liver lesion, which was present in all meerkats to a variable degree, including some with concurrent portal suppurative thrombosis, was acute random multifocal suppurative hepatitis **(**Figs. 1c, 2c**)**. *B. pseudomallei* was cultured from every sample of liver for which culture was pursued **(**Table 1**)**.

Lung lesions were present in all meerkats, and were generally similar, grossly appearing as 1–2 mm pale foci surrounded by red rims, distributed either near the free edges of lung lobes, or diffusely **(**Fig. 1d**)**. Histologically, all lung lesions appeared as multifocal variably-sized random foci of neutrophil infiltration, frequently with surrounding haemorrhage and consistent with bacteraemic spread to the lungs **(**Fig. 2d**)**. Some pulmonary lesions were associated with large thrombosed, necrotic vessels infiltrated with neutrophils. *B. pseudomallei* was cultured from lung in all eight cases. Although *B. pseudomallei* was cultured from every spleen sample in which culture was pursued **(**Table 1**)**, the spleens were grossly normal, and histological findings limited to congestion and patchy increase in neutrophils within red pulp sinuses.

Histological examination of the vertebral column in two meerkats (cases #7 and #8) that clinically exhibited paresis or lameness involving the hind legs, revealed multifocal necrosis and neutrophil infiltration of the bone marrow, in one case with copious suppurative exudation expanding through the cortex into the spinal canal.

Specific bacterial culture aimed at detecting *B. pseudomallei* in samples from the pharyngeal, gastric, rectal, blood, and skin lesions samples from the two surviving meerkats was negative for all samples.

### Environmental sampling

**Fig. 3:**
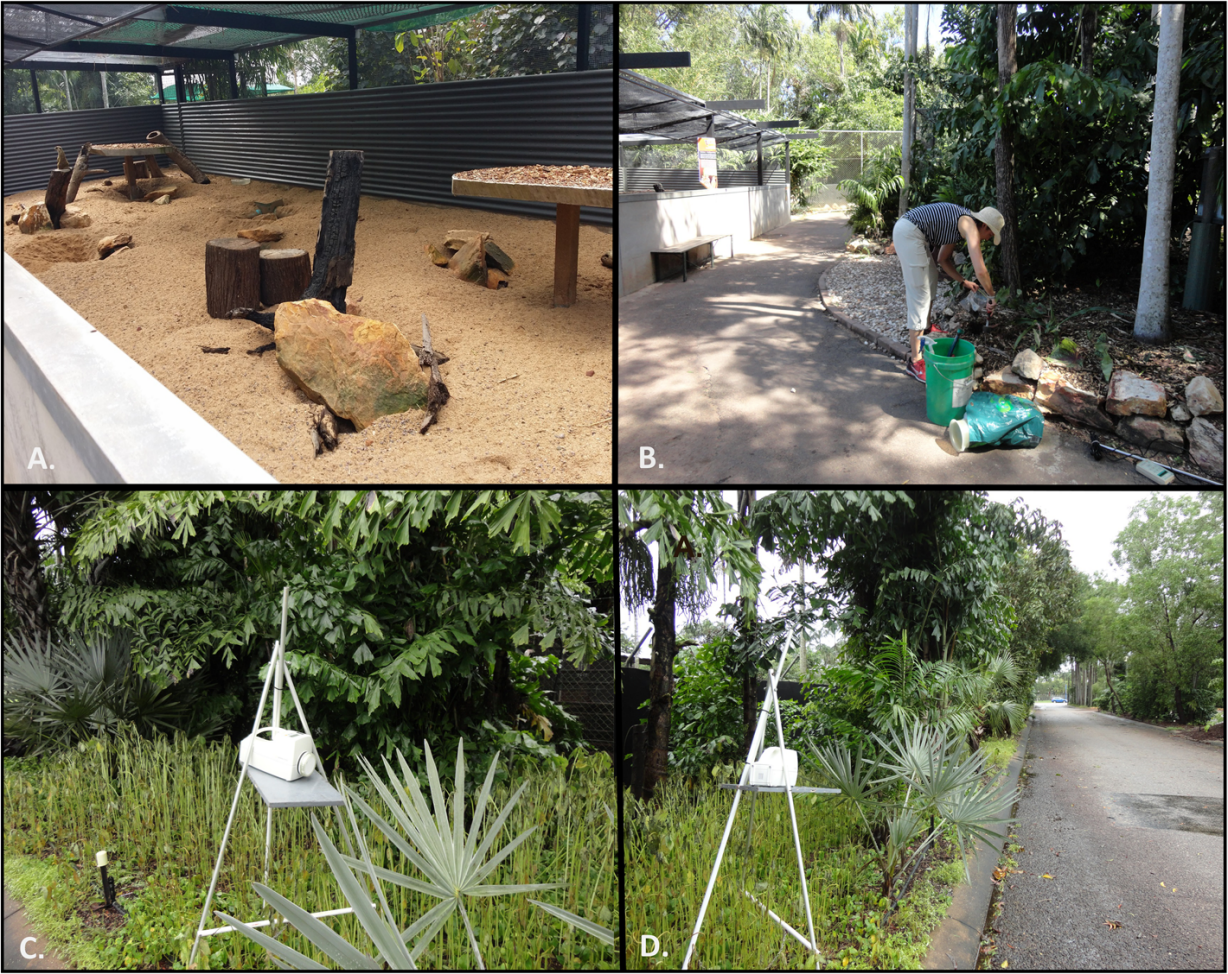
Environmental sampling and isolation of *B. pseudomallei* at the Wildlife Park. Image of meerkat enclosure (**a**) and locations where *B. pseudomallei* was isolated in relation to the case cluster investigation. Image (**b**) displays the garden bed in front of the enclosure where the two positive soil samples were collected, while images (**c**) and (**d**) show the set-up and positioning of the positive air sample collected from the parking lot

*B. pseudomallei* was isolated from three of the 64 environmental samples (3/64, 4.7%) collected at the park as part of the investigation. Two were from separate soil samples isolated from a garden bed in front of the meerkat enclosure and were collected approximately one month apart. The third was isolated from an air sample retrieved from a nature strip along the Wildlife Park parking lot during a low-pressure monsoon trough, less than 100 m from the meerkat enclosure **(**Fig. 3, Additional file 2: Data set S1).

### ST-36 is the strain responsible for infection in all but one meerkat

**Fig. 4:**
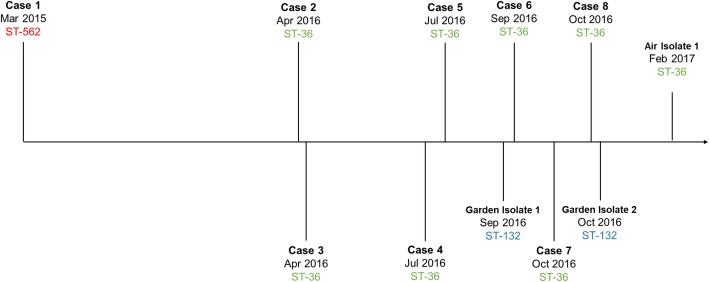
Dates of meerkat deaths and isolation of environmental isolates. Timeline of the eight meerkat melioidosis fatalities and isolation of the three *B. pseudomallei*-positive environmental samples. The ST types of all clinical and environmental isolates are labelled by colour (ST-562, red; ST-36, green; ST-132, blue)

Using the described in-house SNP ST PCR assays, *B. pseudomallei* isolates from seven of the eight (7/8, 87.5%) available meerkat isolates were designated as ST-36 [31]. The isolate from the first meerkat infected at the park (MSHR8750) was classified as ST-562. ST-36 was also assigned to the air sample isolate taken from the Wildlife Park parking lot, while the two soil sample isolates both originating from the garden bed across from the enclosure were designated as ST-132 **(**Fig. 4**)**.

### WGS analysis reveals some clonality between ST-36 meerkat isolates

**Fig. 5:**
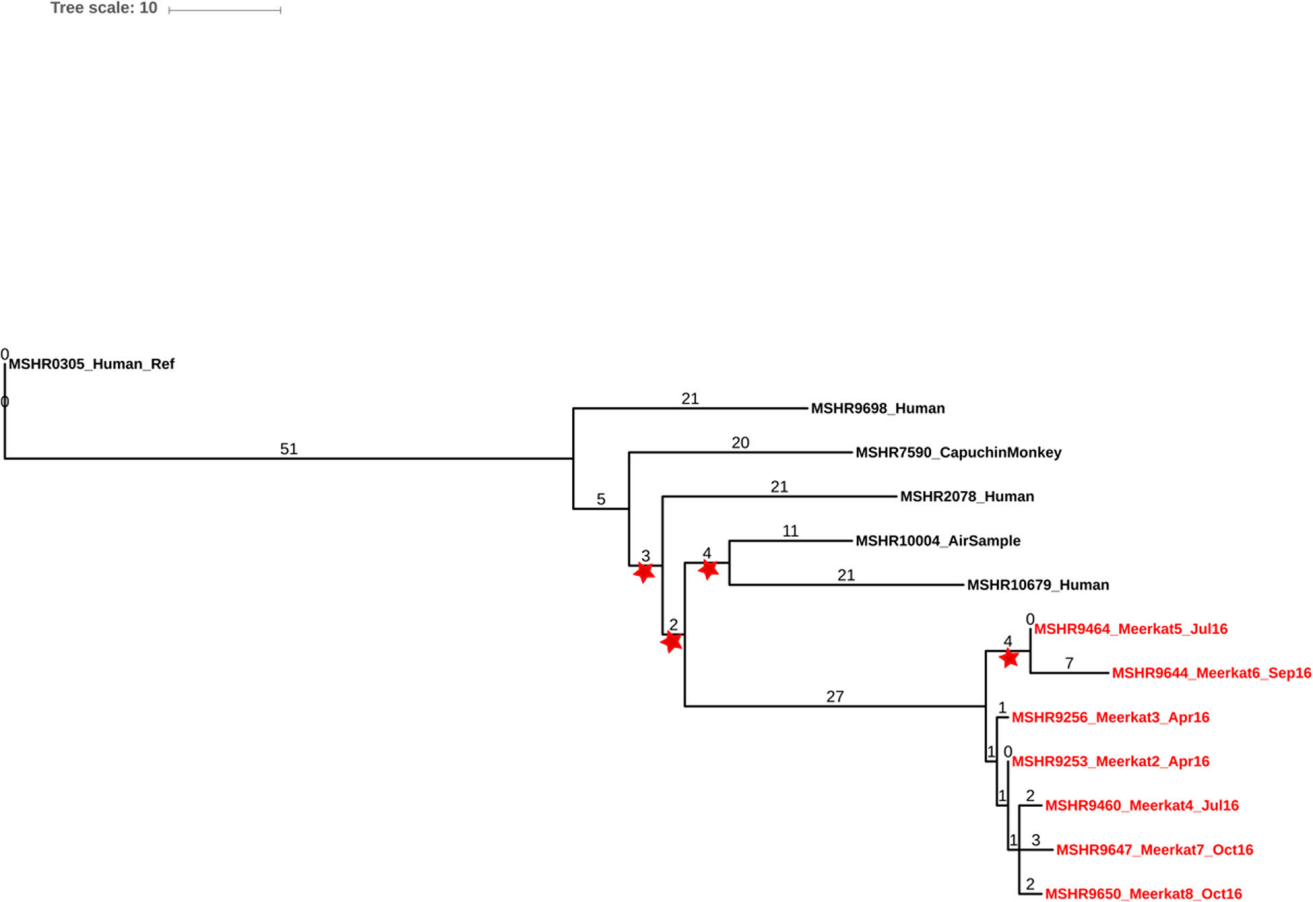
Phylogenetic reconstruction of *B. pseudomallei* ST-36 genomes. Of the eight meerkats, seven with ST-36 *B. pseudomallei* genomes were included in the phylogeny. Maximum parsimony reconstruction of 13 *B. pseudomallei* ST-36 genomes was performed using 194 core-genome orthologous SNPs and InDels (overall consistency index (CI) = 0.9327). Isolates are labelled as described and are denoted by respective isolate (MSHR) ID’s. Red stars on branches denote bootstrap values < 80%

To investigate whether the seven ST-36 meerkats were infected by a single point-source a comparative phylogeny was constructed using WGS data (Fig. 5). Six additional ST-36 human, animal and environmental isolates, two of which had previously been isolated from the same Darwin, Australia Wildlife Park, were included as close references. Maximum parsimony phylogenetic reconstruction of the ST-36 isolates identified 194 total orthologous SNPs and InDels across the 13 genomes. For the seven meerkat isolates, only 22 total orthologous SNP and InDel variants were identified and the isolates clustered closely together on one branch of the tree, indicating they were from the same lineage. Some chronological clustering between five of the isolates (MSHR9253, MSHR9256, MSHR9460, MSHR9647, MSHR9650) was also observed. Additionally, only a single variant was detected between two of the meerkats (MSHR9253; case #2 and MSHR9256; case #3), both of whom died of their infections three days apart in April 2016. Similarly, both fatal cases occurring in October 2016 (MSHR9647; case #7 and MSHR9650; case #8) were infected with closely related *B. pseudomallei* isolates, differing by only five variants. Although the black-capped capuchin isolate (MSHR7590) and air isolate (MSHR10004) were both collected from the same Wildlife Park, they did not cluster with the meerkat isolates and varied by more than 40 orthologous SNPs and InDels from one another.

Lastly, we investigated the presence of the variably present *fhaB3* gene and mutually exclusive *bimA*_*Bp*_ and *bimA*_*Bm*_ genotypes [34]*,* virulence factors used as determinants of geographic origin and pathogenicity [7]. The seven meerkats with ST-36 *B. pseudomallei* infections were designated as carriers of the *bimA*_*Bp*_ gene variant, as was the meerkat with the ST-562 infection (MSHR8750). All eight meerkat isolates including the ST-562 meerkat were also negative for the *fhaB3* virulence gene. This is in contrast to most Australian *B. pseudomallei* isolates overall, but is seen commonly in ST-36 [7]. The four human, one capuchin and one air sampler ST-36 isolates were also negative for the *fhaB3* gene variant.

## Discussion

Unlike many other bacterial infections, melioidosis is not normally considered communicable, with only a small number of zoonotic, human-to-human and animal-to-animal cases reported [2, 35, 36]. Since the eight fatal meerkat *B. pseudomallei* infections occurred over a short time frame, this prompted suspicion of a single contaminated environmental source within the park enclosure. To fully investigate the aetiology of the case cluster, high-resolution WGS and detailed pathology data were combined to identify two separate environmental sources of *B. pseudomallei* infection with suggested variable routes of transmission. Only one of the *B. pseudomallei* strains, ST-36, was implicated in the large case cluster observed in seven of the meerkats. Despite this, extensive sampling efforts at the Wildlife Park failed to reveal the exact origin of the infections, with neither of the two *B. pseudomallei* ST-132 garden bed isolates nor the ST-36 air sample isolate matching any of the eight meerkat genomes. Despite this, our data collectively showed that most of the cases were likely related to a single strain, with gross and histopathological examination suggesting two main routes of infection: cutaneous inoculation and ingestion.

Data from the ST-36 reconstructed phylogeny revealed only minor differences across the seven meerkat strains, with infecting genomes clustering closely together on one branch of the tree. While the ST-36 air sample and black-capped capuchin isolates were also collected from the same Wildlife Park, they did not group together with the meerkat isolates, further indicating that the seven meerkat *B. pseudomallei* strains were epidemiologically related to one another. The *B. pseudomallei* genome has an exceedingly high rate of lateral gene transfer and recombination [29] in its natural soil environment. *B. pseudomallei* can also undergo genetic change due to host selective pressures and these changes can range from small genetic changes caused by mutations to large reductive evolutionary changes [37, 38]. Since the meerkat ST-36 outbreak took place over more than seven-months and samples were isolated at variable time points throughout this period, it could explain why we observed some SNP diversity between isolates. Small SNP variations have been seen in other investigations linking cases of melioidosis to epidemiologically-related environmental *B. pseudomallei* strains previously [23, 33, 39]. Moreover, all isolates were found to contain the *bimA*_*Bp*_ rather than the *bimA*_*Bm,*_ locus variant that is normally associated with more severe neurological manifestations of the disease. The *B. pseudomallei* meerkat isolates were also negative for *fhaB3*, which has been associated with decreased pathogenicity in previous human studies [7]. This may in part explain the relatively slow disease progression observed in the meerkats, in comparison to the rapid sepsis and death seen in other descriptions of melioidosis in imported exotic animals [14, 40].

While *B. pseudomallei* was not isolated from any sand, soil or swab samples collected from within the enclosure, inoculation through the skin was determined as the most likely mechanism of pathogenesis in the majority of cases. Sites of cutaneous infection tended to be centred on sites of previous trauma, such as bony protuberances of the distal limbs, scrotum and tail, suggesting inoculation at sites of skin damage. The deep suppurative skin lesions were the only melioidosis lesions that were associated with fibroplasia, suggesting they were of the longest duration [41], and corroborating that skin inoculation was likely the primary route of infection. As *B. pseudomallei* has been detected in faecal samples of other species including wallabies and chickens [11], it is possible the bacterium was shed into the enclosure by previously infected meerkats and wounds became infected while burrowing or digging around contaminated sand. Likewise, animal-to-animal transmission of the infection may have occurred through lesions and bites sustained from intraspecific aggression among the meerkats. Of note, in an effort to prevent future fighting and decrease stress amongst the remaining meerkats, only males were reintroduced to the enclosure after October 2016, when the final case fatality occurred. Since then no melioidosis infections have been reported in the park meerkats. While animal-to-animal transmission is considered rare, it has been described before in Australian goats and pigs and is believed to occur through wound or nasal secretions, milk, faeces and urine [9, 42]. Accordingly, transmission through wounds inflicted during fighting likely played some role in infection, which is supported by *B. pseudomallei* never having been recovered inside the enclosure.

The second main route of infection suggested by the pathology seen in these cases is ingestion, given the severity and relatively long duration (associated fibroplasia) of lesions specifically involving the hepatic portal vessels that drain pathogens entering from the gastrointestinal tract [43]. Meerkats may have a particular susceptibility to the ingestion route of infection given its apparent relative rarity in some other species [3], although in one other reported outbreak of melioidosis in meerkats portal vascular targeting of lesions was not described [21]. It is possible that the presence of skin lesions harbouring *B. pseudomallei* and infection by ingestion are related, given the grooming habits of meerkats and observation by keepers of the meerkats licking and chewing at skin lesions. Moreover, all food, drinking water and bowl swab samples were culture-negative for the bacterium, though this may have been the result of under-sampling, decontamination of the water bowl, or rotation of the food supply prior to sample collection. Additionally, while detection of the ST-36 air isolate nearby to the enclosure provides support for aerosolised *B. pseudomallei* being passed into the open-air enclosure and potentially contaminating the drinking water or food supply, the air ST-36 was genetically too distant to the meerkat ST-36 isolates to be the infecting strain and this mode of contamination of the enclosure therefore remains only speculative.

Lung lesions in all cases were either random multifocal and/or centred on pulmonary vessels. This is suggestive of bacteraemic embolic spread from the systemic circulation [44] rather than bronchopneumonia from inhalation, which is a common pathogenesis in other species [3, 13, 14, 45, 46]. The occurrence of most of the cases in the drier months (between April and October) rather than the monsoon season also fits with inhalation not being a major source of infection, since inhalational melioidosis has been shown to occur more frequently during severe-weather related events and heavy monsoonal rains [3, 9]. In most of the meerkats there were also random multifocal suppurative hepatic foci, suggesting embolic spread from systemic circulation. These random multifocal lesions in the liver and lung, along with culture of *B. pseudomallei* from the spleen, indicate terminal septicaemia due to *B. pseudomallei* occurred in all the meerkats, a common sequela to infection among many species [6, 10, 46]. There was no pathological evidence of chronicity or latency, such as fibrous nodules within organs in any of the meerkats.

## Conclusions

In the current investigation, we used high-resolution WGS concurrently with detailed pathology data to investigate a cluster of fatal *B. pseudomallei* infections in slender-tailed meerkats. While more traditional *B. pseudomallei* molecular fingerprinting schemes can often confound inferences about infection aetiology and transmission due to the exceedingly high rate of genetic recombination, our findings demonstrate the epidemiological insights that can be gained from high-throughput WGS. Additionally, our data verifies the importance of WGS for expanding the current knowledge of *B. pseudomallei* genotype diversity and point-source attribution in animal cases of melioidosis. These veterinary pathology and genomics findings have important “One Health” implications in providing insights into human melioidosis epidemiology regarding both routes of infection and point-source attribution.

While no environmental point-source was determined, seven of the meerkat strains were highly similar on the whole-genome level, implying that the cases were related. Despite this, pathology data indicated that even amongst the seven clonal ST-36 strains, routes of infection likely varied between the meerkats. Collectively, these findings suggest that meerkats are very susceptible to melioidosis and that stress and intraspecific competition amongst the animals can play an important role in infection. Strategies to reduce the risk of infection are imperative for highly susceptible non-native species imported into melioidosis endemic areas, particularly for iconic exotic species on display in zoos and wildlife parks.

## Methods

### Animals and animal sample processing

The eight deceased slender-tailed meerkats (*Suricata suricatta*) were from a Wildlife Park in the Darwin region (12.5^°^S) of Northern Australia. One female and one male meerkat imported from a Tasmanian zoo in Australia were introduced to the park in late 2014. The index case was the adult male, found dead inside the enclosure in March, 2015. Two additional males were introduced to the park shortly after and two litters totalling eight meerkats were born in mid-2015. Seven subsequent fatal cases of melioidosis occurred in the female matriarch and six of the adolescent meerkats between February and October 2016. For cases #2 and #8, the meerkats were observed unwell for 1–2 days prior to being found dead. Otherwise, clinical signs were observed for 1–2 weeks prior to death, and commonly included lethargy, and in some cases, cutaneous sores, which the meerkats were observed chewing or licking at (cases #3, #4, #5, #8), and limping or hind limb paresis (cases #3, #7, #8). Several meerkats that were observed to be unwell were treated with 10 mg/kg doxycycline administered orally in food treats once daily (Psittavet, Vetafarm, Wagga Wagga, NSW). Two cases were treated with cefovexin, 8 mg/kg injected subcutaneously (Convenia, Zoetis, Rhodes, NSW); case #2 was treated twice, once at five weeks and again four days prior to death, and case #3 was treated one day prior to death. Treated animals were allowed to remain with the colony to minimise stress.

Complete gross post-mortem examinations were performed on the eight deceased meerkats at Berrimah Veterinary Laboratories (BVL). For histology, tissue samples were fixed in 10% phosphate buffered formalin, processed in standard fashion and stained with haematoxylin and eosin. A wide range of tissues were examined histologically for each meerkat, including liver, lung, spleen, heart, kidney and brain, plus stomach and multiple sections of intestine in the better-preserved carcasses, and bones or skin in selected carcasses in which the clinical history suggested possible involvement or where gross lesions were observed.

Post-mortem samples for bacterial culture were collected aseptically and selected case by case based on whether the tissue exhibited a gross lesion or could add to information on pathogenesis **(**Table 1**)**. For aerobic bacterial culture swabs of lesions or tissue samples were homogenised with physiological saline, placed in Ashdown broth and incubated at 35 °C for seven days for enrichment culture. Samples were then plated onto Tryptic soy agar with sheep’s blood, MacConkey and Ashdown agars (Thermo Fisher Scientific, Thebarton, Australia) and incubated at 35 °C for 48 h (Additional file 1: Fig. S1). Isolates were identified using the API 20 NE system (bioMérieux, Marcy l’Etoile, France). Those classified as *B. pseudomallei* were transferred to Menzies School of Health Research (Menzies) for further confirmation and strain designation.

In addition to post-mortem samples, samples were taken under general anaesthetic from two meerkats that were in the colony and survived the outbreak to determine if they might be subclinical carriers of *B. pseudomallei*. Samples included gastric aspirates, throat and rectal swabs. All were placed directly into Ashdown broth and blood was also placed directly into thioglycollate broth.

### Environmental sample collection

Six rounds of environmental sampling were carried out at the park between May 2016 and March 2017 in relation to the meerkat fatalities to ensure thorough sampling of the enclosure and all adjacent park areas. Samples were collected from within the meerkat enclosure, nearby animal enclosures and adjacent garden areas. A total of 64 samples were collected from the park, including 33 soils, five waters, nine environmental swabs, five plants, two food, three faecal samples, and seven air samples collected using a portable high-volume air sampler with gelatin membrane filters (Sartorius MD8, Goettingen, Germany) (Additional file 2 Data set S1).

### Environmental sample processing and confirmation

Culture of *B. pseudomallei* from water, soil, air and swab specimens was carried out using methods previously developed in our laboratory [23, 27, 47]. Briefly, samples were enriched in Ashdown’s broth containing colistin (50 mg/L) and incubated at 37 °C aerobically for 2 and 7 days. Enriched broth was plated onto Ashdown’s agar with gentamicin (8 mg/L) and incubated for 48 h, and all colonies resembling *B. pseudomallei* were sub-cultured onto Ashdown’s agar. DNA from suspected colonies was extracted using 10% Chelex-100 resin [48] and confirmation of *B. pseudomallei* was carried out using a real-time PCR assay targeting a 115 bp segment within the type three secretion system 1 (TTS1) gene cluster [49] specific to *B. pseudomallei*.

### Isolates used for comparative phylogenomic analysis

*B. pseudomallei* genomes from 13 ST-36 isolates originating from Darwin, Australia were used in the analysis **(**Additional file 3: Data set S2). Isolates were collected between 1994 and 2017. Eight animal isolates were used in the reconstructed phylogeny: the seven ST-36 meerkat isolates from the outbreak (MSHR9253, MSHR9256, MSHR9460, MSHR9464, MSHR9644, MSHR9647, MSHR9650); and one isolate from a black-capped capuchin (*Sapajus apella*) (MSHR7950). The black-capped capuchin was an exotic import on display at the same Wildlife Park and died from melioidosis in 2012. Also included in the ST-36 phylogenetic analysis were one air sample (MSHR10004) collected as part of the park environmental survey and four isolates from human infections collected as part of the Darwin Prospective Melioidosis Study (Human Research Ethics Committee of the Northern Territory Department of Health and the Menzies School of Health Research, approval HREC 02–38) [3] (MSHR0305, MSHR2078, MSHR9698, MSHR10679) (Additional file 3: Data set S2).

### Whole-genome sequencing of clinical and environmental Wildlife Park isolates

Genomic DNA was extracted using the Qiagen DNeasy blood and tissue kit (Qiagen, Chadstone, Victoria, Australia) as previously described [24]. Isolates were sequenced at Macrogen, Inc. (Gasan-dong, Seoul, Republic of Korea) or Australian Genome Research Facility Ltd. (Melbourne, Australia) using the Illumina HiSeq2000 and Illumina HiSeq2500 platforms (Illumina, Inc., San Diego, CA).

Identification of orthologous core genome single-nucleotide polymorphism (SNP) and small insertion or deletion (InDel) variants from WGS data was performed using SPANDx [50]. MSHR0305, an isolate obtained from a Darwin melioidosis patient in 1994, was used as a high-quality ST-36 reference assembly (N50: 4054155, total length: 7428072, contigs: *n* = 2). Phylogenetic reconstruction was based on maximum-parsimony in PAUP v4.0a162 [51] using both SNP and InDel variants to increase resolution [33]. Bootstrapping with 1000 replicates was carried out to determine robustness of branches. Phylogenetic trees were examined in FigTree v1.4.4 (http://tree.bio.ed.ac.uk/software/figtree/) and managed using the online tool iTOL v3: Interactive Tree of Life [52].

### Multi-locus sequence typing (MLST) assignment

MLST types for human clinical isolates (*n* = 4) were assigned from WGS data in silico prior to the study commencing using the BIGSdb tool accessible on the *B. pseudomallei* MLST website (http://pubmlst.org/bpseudomallei/) [28, 53]. MLST assignment of *B. pseudomallei* meerkat isolates (*n* = 8), black-capped capuchin isolate (*n* = 1) and environmental isolates collected as part of the park survey (*n* = 3) was done utilising a series of in-house PCR primers targeting the common Darwin ST types 36, 109, 132 and 562 [31]. ST types were later confirmed using WGS data assigned in silico*.*

### Virulence factor assignment

Virulence factor *fhaB3* and *bimA*_*Bm/Bp*_ positivity were determined using the online basic local alignment search tool (BLAST) as previously described [54]. In brief, the genes were BLAST searched against the *B. pseudomallei* WGS using the nucleotide BLAST (BLASTn) parameter, and each genome was assigned as *fhaB3* (encoded by *BPSS2053* in *B. pseudomallei* K96243) positive or negative, and as a carrier of *BimA*_*Bm*_ (*BURPS668_A2118* in *B. pseudomallei* MSHR668) or *BimA*_*Bp*_ (*BPSS1492* in *B. pseudomallei* K96243).

## Supplementary information

**Additional file 1: Figure S1.** Clinical microbiological detection of *B. pseudomallei* in meerkats *B. pseudomallei* growth on Sheep’s Blood Agar (A.), and growth on selective Ashdown Agar (B.) Gram-negative bacilli characteristic of *B. pseudomallei* as seen by Gram-stain (C.) (bar = 10 μm)

**Additional file 2: Data set S1.** Data set 1**-** Environmental samples collected at the Wildlife Park in response to the meerkat case cluster

**Additional file 3 Data set S2.** Data set 2**-** ST-36 isolates used for WGS phylogenetic comparison

## Data Availability

The dataset supporting the conclusions of this article is available in the National Center for Biotechnology Information Short Read Archive, under BioProject accession number PRJNA532306 (https://www.ncbi.nlm.nih.gov/bioproject/532306), accessions SAMN11394028-SAMN11394038.

## Abbreviations

*B. pseudomallei*: *Burkholderia pseudomallei*
BVL: Berrimah Veterinary Laboratories
InDel: Insertion and/or deletion
MLST: Multi-locus sequence type
NT: Northern Territory
SNP: Single nucleotide polymorphism
ST: Sequence type
WGS: Whole-genome sequencing
