# Supplementary material for: Melioidosis fatalities in captive slender-tailed meerkats (Suricata suricatta): combining epidemiology, pathology and whole-genome sequencing supports variable mechanisms of transmission with one health implications
Source: BMC Vet Res. 2019 Dec 19;15:458. doi: 10.1186/s12917-019-2198-9 (PMC6921467; doi:10.1186/s12917-019-2198-9)
Supplement: Supplementary file 1 — Additional file 1: Figure S1. Clinical microbiological detection of B. pseudomallei in meerkats B. pseudomallei growth on Sheep’s Blood Agar (A.), and growth on selective Ashdown Agar (B.) Gram-negative bacilli characteristic of B. pseudomallei as seen by Gram-stain (C.) (bar = 10 μm) [file 12917_2019_2198_MOESM1_ESM.pdf]

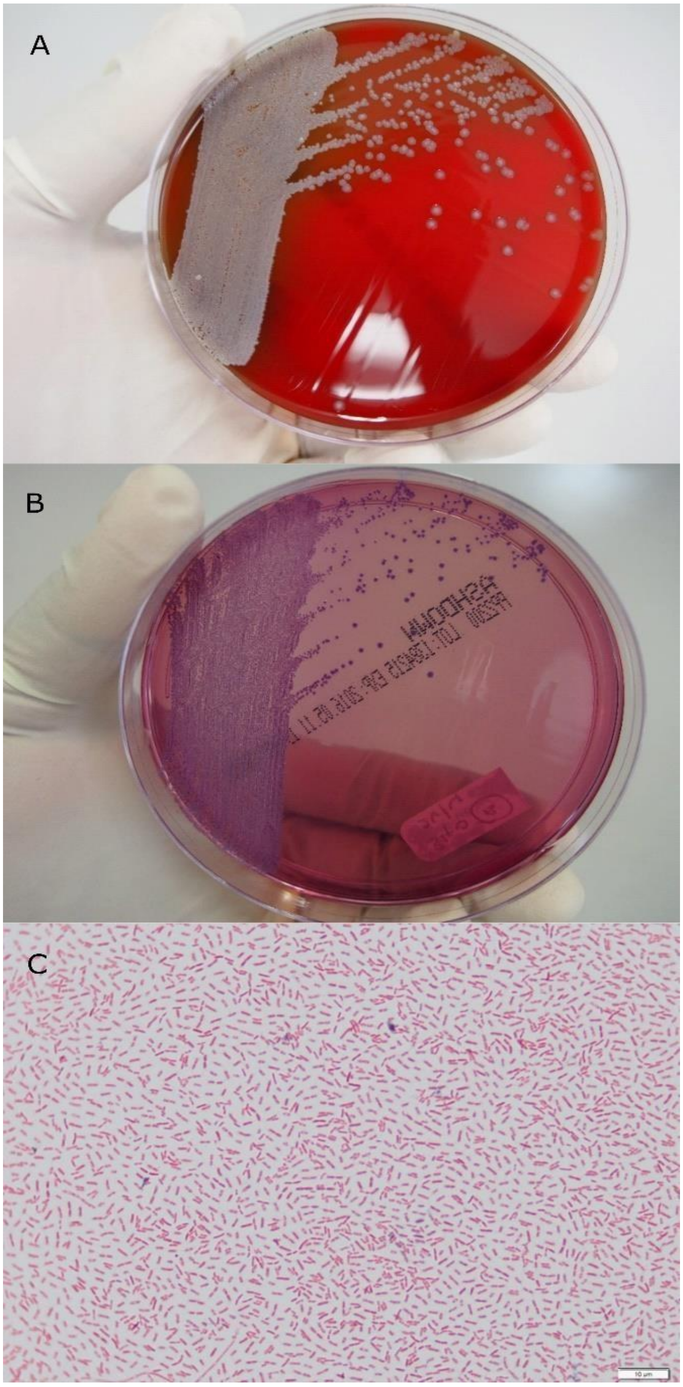

Supplementary data- Figure S1- Clinical microbiological detection of *B. pseudomallei* in meerkats *B. pseudomallei* growth on Sheep's Blood Agar **(A.)**, and growth on selective Ashdown Agar (**B.)** Gramnegative bacilli characteristic of *B. pseudomallei* as seen by Gram-stain (**C**.) (bar = 10µm).
