# Supplementary material for: Melioidosis fatalities in captive slender-tailed meerkats (Suricata suricatta): combining epidemiology, pathology and whole-genome sequencing supports variable mechanisms of transmission with one health implications
Source: BMC Vet Res. 2019 Dec 19;15:458. doi: 10.1186/s12917-019-2198-9 (PMC6921467; doi:10.1186/s12917-019-2198-9)
Supplement: Supplementary file 2 — Additional file 2: Data set S1. Data set 1- Environmental samples collected at the Wildlife Park in response to the meerkat case cluster [file 12917_2019_2198_MOESM2_ESM.docx]

**Additional file 1- Data set 1-** Environmental samples collected at the Wildlife Park in response to the meerkat case cluster.

| **Sample ID** | **Sample type** | **Date of collection** | **Sample location** | ***B. pseudomallei* isolated** |
| --- | --- | --- | --- | --- |
| CP23 | Soil | 12/05/2016 | Meerkat enclosure sandy soil, near gate | No |
| CP24 | Soil | 12/05/2016 | Meerkat enclosure sandy soil, opposite side to gate | No |
| CP25 | Soil | 12/05/2016 | Meerkat enclosure sandy soil, furthest corner from gate | No |
| CP26 | Soil | 12/05/2016 | Meerkat enclosure sandy soil, opposite corner to CP25 | No |
| CP27 | Soil | 12/05/2016 | Meerkat enclosure sandy soil, in ground burrow | No |
| CP28 | Soil | 12/05/2016 | Meerkat enclosure sandy soil, in ground burrow | No |
| CP29 | Soil | 12/05/2016 | Meerkat enclosure sandy soil, under table where food kept | No |
| CP30 | Soil | 12/05/2016 | Soil outside of meerkat enclosure | No |
| CP31 | Soil | 12/05/2016 | Soil outside of meerkat enclosure | No |
| CP32 | Soil | 12/05/2016 | Soil outside of meerkat enclosure | No |
| CP33 | Soil | 12/05/2016 | Soil collected next to water runoff area in Timor pony enclosure | No |
| CP34 | Soil | 12/05/2016 | Timor pony enclosure | No |
| CP35 | Soil | 12/05/2016 | Garden bed in front of meerkat enclosure | No |
| CP36 | Soil | 12/05/2016 | Garden bed in front of meerkat enclosure | No |
| CPW1 | Water | 12/05/2016 | Water collected from Timor pony enclosure | No |
| CPA1 | Air | 12/05/2016 | Air facing meerkat enclosure | No |
| CPA2 | Air | 12/05/2016 | Air facing Timor pony enclosure adjacent to meerkats | No |
| CP38 | Soil | 27/09/2016 | Garden bed opposite meerkat enclosure | **Yes** |
| CP39 | Soil | 27/09/2016 | Inside meerkat enclosure front left corner | No |
| CP40 | Soil | 27/09/2016 | Inside meerkat enclosure front centre | No |
| CP41 | Soil | 27/09/2016 | Inside meerkat enclosure right corner | No |
| CP42 | Soil | 27/09/2016 | Inside meerkat enclosure back right corner | No |
| CP43 | Soil | 27/09/2016 | Soil sample from under bamboo plant behind meerkat enclosure | No |
| CPW20 | Water | 27/09/2016 | Tap near rear of meerkat enclosure, dam water used on side garden | No |
| CPW21 | Water | 27/09/2016 | Town water tap opposite meerkat enclosure, used for drinking water and pen cleaning | No |
| CPA4 | Air | 27/09/2016 | Air sampled on top of Meerkat enclosure facing north | No |
| CPM1 | Food | 27/09/2016 | Mealworms, meerkat feed | No |
| CPM2 | Food | 27/09/2016 | Mealworms, meerkat feed | No |
| CPM3 | Plant | 27/09/2016 | Bamboo leaves at back of meerkat enclosure | No |
| CPM4 | Plant | 27/09/2016 | Bamboo stems at back of meerkat enclosure | No |
| CPM5 | Faeces | 27/09/2016 | Faeces from meerkat enclosure | No |
| CPSW25 | Env. swab | 30/09/2016 | Water bowl swab inside enclosure | No |
| CPSW26 | Env. swab | 30/09/2016 | Water bowl swab inside enclosure | No |
| CPSW27 | Env. swab | 30/09/2016 | Inside den box in enclosure | No |
| CPSW28 | Env. swab | 30/09/2016 | Inside log in enclosure | No |
| CP44 | Soil | 20/10/2016 | Soil from under hole cut in plastic ground cover in middle of enclosure | No |
| CP45 | Soil | 20/10/2016 | Soil from under hole cut in plastic ground cover in the back of the enclosure | No |
| CP46 | Soil | 20/10/2016 | Soil from inside burrow in middle of enclosure | No |
| CP47 | Soil | 20/10/2016 | Soil from under bamboo at back of enclosure near gate | No |
| CP48 | Soil | 20/10/2016 | Soil from garden bed at front of enclosure | **Yes** |
| CP49 | Soil | 20/10/2016 | Soil under enclosure output pipe | No |
| CP50 | Soil | 20/10/2016 | Soil under enclosure output pipe | No |
| CP51 | Soil | 20/10/2016 | Soil from garden bed at side of enclosure | No |
| CPW23 | Water | 20/10/2016 | Tap near rear of meerkat enclosure, dam water not used for meerkats but used on side garden | No |
| CPW24 | Water | 20/10/2016 | Water from runoff pond next to Timor pony enclosure | No |
| CPSW29 | Env. swab | 20/10/2016 | Swab from hollow log inside enclosure | No |
| CPSW30 | Env. swab | 20/10/2016 | Swab of table where sit at left side of enclosure | No |
| CPSW31 | Env. swab | 20/10/2016 | Swab under log at front of enclosure | No |
| CPSW32 | Env. swab | 20/10/2016 | Swab of tunnels inside burrow | No |
| CPSW33 | Env. swab | 20/10/2016 | Swab from inside burrow at back of enclosure | No |
| CPM6 | Plant | 20/10/2016 | Dried bamboo leaves inside enclosure | No |
| CPM7 | Plant | 20/10/2016 | Black bamboo stalk and leaves at back of enclosure near gate | No |
| CPM8 | Plant | 20/10/2016 | Yellow bamboo stalk and leaves at back of enclosure | No |
| CPM9 | Faeces | 20/10/2016 | Timor pony faeces | No |
| CPM10 | Faeces | 20/10/2016 | Meerkat faeces | No |
| CPA5 | Air | 20/10/2016 | Air from back of enclosure facing Timor ponies | No |
| CPA6 | Air | 20/10/2016 | Air from front of enclosure facing garden bed | No |
| CPA7 | Air | 6/02/2017 | Air from garden on right side of parking lot as walking into park, approximately 100 metres from meerkat enclosure | **Yes** |
| CPA8 | Air | 6/02/2017 | Air from along grassy bank next to park fence | No |
| CP52 | Soil | 1/03/2017 | Soil along grassy bank next to park fence, where air sample came positive | No |
| CP53 | Soil | 1/03/2017 | Soil along grassy bank next to park fence, where air sample came positive | No |
| CP54 | Soil | 1/03/2017 | Soil along grassy bank next to park fence, where air sample came positive | No |
| CP55 | Soil | 1/03/2017 | Soil along grassy bank next to park fence, where air sample came positive | No |
| CP56 | Soil | 1/03/2017 | Soil along grassy bank next to park fence, where air sample came positive | No |
