# Supplementary material for: Melioidosis fatalities in captive slender-tailed meerkats (Suricata suricatta): combining epidemiology, pathology and whole-genome sequencing supports variable mechanisms of transmission with one health implications
Source: BMC Vet Res. 2019 Dec 19;15:458. doi: 10.1186/s12917-019-2198-9 (PMC6921467; doi:10.1186/s12917-019-2198-9)
Supplement: Supplementary file 3 — Additional file 3 Data set S2. Data set 2- ST-36 isolates used for WGS phylogenetic comparison [file 12917_2019_2198_MOESM3_ESM.docx]

| **Sample ID** | **Original species designation** | **Source of sample** | **Year** | **Region of sample origin (NT, Aus)** | **GenBank/SRA accession no.** | **Reference** |
| --- | --- | --- | --- | --- | --- | --- |
| MSHR0305 | *Burkholderia pseudomallei* | Human | 1994 | Rural Darwin | CP006469, CP006470 | Stone et al., Genome Announc, 2013, 1(4):e00656-13 |
| MSHR2078 | *Burkholderia pseudomallei* | Human | 2005 | Urban Darwin | SAMN04226283 | Price EP et al., Appl Environ Microbiol, 2015, 25;82(3):954-63 |
| MSHR7590 | *Burkholderia pseudomallei* | Black-capped capuchin | 2012 | Urban Darwin | SAMN11394028 | This publication |
| MSHR9253 | *Burkholderia pseudomallei* | Meerkat | 2015 | Urban Darwin | SAMN11394029 | This publication |
| MSHR9256 | *Burkholderia pseudomallei* | Meerkat | 2016 | Urban Darwin | SAMN11394030 | This publication |
| MSHR9460 | *Burkholderia pseudomallei* | Meerkat | 2016 | Urban Darwin | SAMN11394031 | This publication |
| MSHR9464 | *Burkholderia pseudomallei* | Meerkat | 2016 | Urban Darwin | SAMN11394032 | This publication |
| MSHR9644 | *Burkholderia pseudomallei* | Meerkat | 2016 | Urban Darwin | SAMN11394033 | This publication |
| MSHR9647 | *Burkholderia pseudomallei* | Meerkat | 2016 | Urban Darwin | SAMN11394034 | This publication |
| MSHR9650 | *Burkholderia pseudomallei* | Meerkat | 2016 | Urban Darwin | SAMN11394035 | This publication |
| MSHR9698 | *Burkholderia pseudomallei* | Human | 2016 | Urban Darwin | SAMN11394036 | This publication |
| MSHR10004 | *Burkholderia pseudomallei* | Environmental (Air) | 2017 | Urban Darwin | SAMN11394037 | This publication |
| MSHR10679 | *Burkholderia pseudomallei* | Human | 2017 | Rural Darwin | SAMN11394038 | This publication |

**Additional file- Data set 2-** ST-36 isolates used for WGS phylogenetic comparison.
